# Answer to Dr. Drake’s Interrogatories

**Published:** 1854-01

**Authors:** 


### DENTAL REGISTER OF THE WEST.

VOL. VII. JANUARY, 1853. No. 2.

### Original Communications.

*Answer to Dr. Drake's Interrogatories.* [Concluded.]

In our last, we replied to your questions in relation to the effect of repeated salivation on the teeth and gums, the effect of tobacco on the teeth, and the origin of salivary calculus.

The fourth question propounded for consideration is—"Is the decay of teeth greater in the West than in the Atlantic States, in the same latitudes; and is there any difference in different latitudes, in the same meridian?

In the answer given to the first question propounded in your letter, we gave a general consideration of the cause of decay in the dental organs, and from the view then taken it would appear that that locality which most effectually secures a perfect constitutional development would necessarily give the best teeth. Different nations are characterized by different physiognomic and constitutional temperaments. The latter (and sometimes the former, when it gives an expanded alveolar arch) may exert, as we have shown, a marked influence on the development of the teeth.

Climate, in so far as it may exert an influence in the formation of a peculiar constitutional diethesis, would hence exert an influence on the dental organism. We have already referred to the effect of diet.

The above influences combined may present a very different dental development, and we have no doubt, in a few generations, very materially modify the general constitutional development. The population of our Atlantic and Western States are so homogenous, and that of the Western States so little of a fixed nature that a definite reply to your inquiry can not well be given.

We are aware that some in the profession have entertained the opinion that some portions of the United States present a marked difference in the perfection of the dental organism. In so far as this may be the case, we are satisfied it can all be traced to the general health of the locality, diet and care. We have, for over twenty years, been a somewhat close observer in the West and South, and we have never been enabled to notice any difference which we could not trace to these causes. We have seen, on the western slopes of the Allegheny, on the broad prairies of the West, and the low, alluvial lands of the Mississippi, the same tendency to decay manifested. In all these localities, in healthy children, with a perfect general constitutional development—their innate constitutions being good—their dental organs generally bear marks of this. The continued tide of eastern immigration west enables us to see daily evidences of the same things. We have often been enabled to trace, in eastern as well as western families, a good denture to the direct interposition of some skillful operator.

A residence on the top of the Allegheny mountain, which would secure health to the mother and child, with a constitution to the latter, would, as we think, give a more perfect denture to that child than a residence in an unhealthy locality, where neither mother nor child could enjoy uninterrupted health.

But as this subject has been elucidated at some length in answer to the first interrogatory, we shall not dwell on it at present. When we take into consideration the chemical organization of the teeth and the elements of the atmosphere itself, we can not suppose that this, whether it be impregnated with miasmatic poison in the low lands of our valleys, or pure as the mountain breeze, would of itself have any direct chemical agency on these compact organs.

That there is a greater tendency in the West to decay of the teeth than in the East, we think needs more testimony to confirm than the profession at present can command. In the United States there appears ever an abundance on which to test the capability of the dental organs, yet in all sections there are multitudes who find more to eat than they can well masticate.

Your fifth inquiry is—"Is there any difference as to soundness of teeth between our native and foreign population?"

This question may be answered in the affirmative, and yet, taken in the broad sense in which it would be received, it would not answer the question satisfactorily. Different nations have, from constitutional development, habits, diet, &c., differently constituted dental organs. Some, for instance the Scotch, live much on that kind of diet which most effectually secures a good osseous system. The oat meal used so very generally in Scotland contains much of the "bran," and this affords a large amount of earthy elements which enter into the teeth. But were we to select any particular nation as having particularly good teeth, we should not specially take the Scotch, and much less the Irish, who also live on that kind of food best adapted to give a perfect denture. But both of these, as well as the natives of the British Isle, have, when first emigrating to this country, better teeth than our native-born citizens, and either of these would compare more favorably than the Irish. Our observation has led us to believe that the French, the Swiss, the Scotch and English have better teeth generally than the Americans, while, if we compare the teeth among the Irish and Americans, taking the same general temperaments, there is no special difference perceptible. The Germans, so far as our observation has extended in Cincinnati, will not show better dentures than our native-born.

It is said by some that our Indian tribes, before commingling with the whites, had but little, if any, decay of their teeth. If this be the case, their association with the whites has very soon brought with it decay of the teeth.

In making these comparisons, it should be borne in mind that, unless we take the same general constitution and temperament, as well as mode of living, the comparison will not be just.

The Brahmins of India, who are said to have the finest teeth in the world, have a custom in cleaning their teeth—which is done daily—which more effectually prevents decay than any other. They use a soft twig, the fibres of which act as a most admirable brush between the teeth, where decay most frequently commences.

Our own population very soon becomes blended with the various races that emigrate to this country, and hence we soon lose our own peculiar physiognomic characteristics, and this is equally so when we come to examine the dental organs.

In relation to your sixth interrogatory, we would remark that Dr. Berry has been for several years engaged in practice in the South, and has made many careful observations, the results of which agree so well with our own observations that we propose giving his answer to the question, which exhibits the tendency to decay which had manifested itself at different periods of life, from fifteen to one hundred years. With this we close our reply to the interrogatories propounded, and would express a regret that we have not been able to give some statistical information in relation to the fourth and fifth of your interrogatories. We have not been able to get this, in that reliable shape which we feel is necessary, and hence must omit it for the present, hoping at some future time to be able to do so.

In answer to your question—"Are the teeth of our colored people less subject to decay than those of white, who labor and live in a similar manner, as to diet and drinks?"—Dr. Berry remarks:

In reference to this question, I resolved several years ago to examine the teeth of a few hundred whites and an equal number of blacks, in Mississippi. After progressing considerably with the examination, it was confined to the blacks, since a knowledge of the comparative amount of decay of the teeth of the two classes, would not be a criterion in deciding the question, on account of the great difference in their regimen.

The examinations were made almost exclusively among field hands, including all at the various quarters visited for this purpose, above fifteen years of age, with the exception of mulattos, quadroons, and cooks. While consanguinity with the whites improves the mental endowments of the colored race, it produces generally a deterioration of their physical organization. Hence, we usually find mulattos and quadroons with feeble constitutions, and their teeth much affected by caries in early life. Decay of the teeth almost invariably, prevails to a greater extent among colored cooks, than among those employed in other occupations.

Among the predisposing causes of dental caries among the negroes of the south, may be mentioned the defective organization of their teeth; the total neglect of cleanliness, so far as they are concerned, and the unhealthiness of the climate. According to an analysis of the teeth by Berzelius, the enamel contains 85.3 parts in the hundred of phosphate of lime, 3.2 fluate of lime, and 8 carbonate of lime, and the dentine or bony substance of the teeth, 62 of phosphate of lime, 2 fluate of lime, and 5.5 carbonate of lime; and as the material for the formation of the teeth is derived from the blood, and that from the food and drink, and the principal food of the negroes of the south generally being swine's flesh and Indian corn, which are deficient in lime, it might readily be inferred, that their teeth would be defective as to a proper amount of this necessary ingredient, and on this account much more liable to be attacked by caries. The tooth brush andother appliances for keeping the teeth free from foreign substances never being used by them, particles of food are constant ly left in the interstices of their teeth, where its decomposition can hardly fail to induce caries to a great extent. During the sickly season they are exposed to attacks of intermittent and other fevers, when the saliva becoming acidified, probably is a fruitful source of caries in the dental organs.

I suppose three-fourths of those whose teeth were examined were natives of Mississippi; and a majority of them were on large plantations, a number of which were on bottom lands, in Hinds and Warren counties, while the others were on upland; so that the table annexed as the result of my examinations presents, probably, a fair criterion as to the condition of the teeth of the negroes generally, in the Southwestern States. The inspection was carefully made, and the least perceptible caries, together with teeth that had been removed marked as such, while full sets of sound teeth were denoted by a cypher. Their ages were in most instances a matter of conjecture, but no doubt tolerably correct.

I think the teeth of the negroes in the south decay as much as those of the whites, and that were they to labor and live as to diet and drinks, in a manner similar to the whites, with equal care as to cleanliness of the mouth, they would suffer from dental caries, at least in an equal degree with them. The bicuspidati and molars of the negroes appear to be more subject to caries than the corresponding teeth of the whites, while the reverse is the case with their incisors and cuspidati, they being less crowded from the greater elongation of their maxillary bones.

Caries in the teeth of colored persons, is mostly the soft, light colored kind, and I think, usually advances more rapidly, than is common with the whites.

The following table is a copy of the notes made, as to the decay and loss of the teeth of each one examined; for example, in the first column for females, from 15 to 25 years of age. The first one examined had but one tooth decayed or lost, the second but one also, the third four, the fourth eight, while the eleventh had a full set of sound teeth, as indicated by a cypher.

### MALES.

*Number of Teeth caries or lost by each one examined.*

| YEARS OF AGE. | | | | | | | | | |
|---|---|---|---|---|---|---|---|---|---|
| 15–20 | 20–25 | 25–30 | 30–35 | 35–40 | 40–45 | 45–50 | 50–55 | 55–60 | 60–100 |
| 1 | 9 | 3 | 11 | 9 | 4 | 0 | 2 | 17 | 9 |
| 2 | 5 | 13 | 2 | 12 | 0 | 15 | 18 | 5 | 26 |
| 2 | 0 | 1 | 2 | 10 | 6 | 10 | 17 | 10 | 8 |
| 3 | 3 | 13 | 14 | 8 | 5 | 19 | 8 | 32 | 10 |
| 0 | 8 | 4 | 5 | 6 | 4 | 20 | 1 | 11 | 20 |
| 5 | 5 | 6 | 0 | 25 | 7 | | 22 | 7 | 20 |
| 1 | 3 | 16 | 3 | 8 | 17 | | 10 | | |
| 1 | 1 | 0 | 1 | 28 | 18 | | 24 | | |
| 4 | 2 | 14 | 2 | 19 | 1 | | 9 | | |
| 0 | 0 | 13 | 1 | 20 | | | | | |
| 3 | 7 | 5 | 4 | 9 | | | | | |
| 1 | 1 | 15 | 15 | 27 | | | | | |
| 3 | 8 | 10 | 7 | 26 | | | | | |
| 2 | 3 | 10 | 11 | 23 | | | | | |
| 2 | 10 | 6 | 2 | 12 | | | | | |
| 2 | 8 | 19 | 9 | 17 | | | | | |
| 3 | 7 | 8 | 8 | 9 | | | | | |
| 8 | 20 | 10 | 10 | 10 | | | | | |
| 7 | 0 | 5 | 11 | 0 | | | | | |
| 13 | 2 | 10 | 6 | | | | | | |
| 12 | 2 | 26 | | | | | | | |
| 4 | 13 | 18 | | | | | | | |
| 7 | 16 | 12 | | | | | | | |
| 2 | 0 | 15 | | | | | | | |
| 4 | 5 | 3 | | | | | | | |
| 4 | 25 | 11 | | | | | | | |
| 1 | 0 | 12 | | | | | | | |
| 3 | 2 | 2 | | | | | | | |
| 2 | 6 | 5 | | | | | | | |
| 0 | 11 | 6 | | | | | | | |
| 22 | 3 | 27 | | | | | | | |
| 5 | 1 | 8 | | | | | | | |
| 8 | 6 | 23 | | | | | | | |
| 2 | 27 | 2 | | | | | | | |
| 5 | 14 | 3 | | | | | | | |
| 4 | 19 | 10 | | | | | | | |
| 4 | 15 | 8 | | | | | | | |
| 5 | 8 | 7 | | | | | | | |
| | 0 | 4 | | | | | | | |
| | 2 | 5 | | | | | | | |
| | 2 | 10 | | | | | | | |
| | 7 | | | | | | | | |
| | 20 | | | | | | | | |
| | 4 | | | | | | | | |
| | 10 | | | | | | | | |
| | 12 | | | | | | | | |
| | 5 | | | | | | | | |
| | 2 | | | | | | | | |

### FEMALES.

*Number of Teeth caries or lost by each one examined.*

YEARS OF AGE.

| 15-20 | 20-25 | 25-30 | 30-35 | 35-40 | 40-45 | 45-50 | 50-55 | 55-60 | 60-100 |
|---|---|---|---|---|---|---|---|---|---|
| 1 | 4 | 11 | 15 | 13 | 1 | 9 | 21 | 32 | 8 |
| 1 | 6 | 8 | 7 | 3 | 6 | 15 | 16 | 17 | |
| 4 | 22 | 14 | 3 | 14 | 18 | 13 | 12 | 23 | |
| 8 | 15 | 11 | 32 | 8 | 7 | 24 | 25 | 29 | |
| 6 | 4 | 10 | 8 | 9 | 22 | 11 | 7 | 22 | |
| 6 | 6 | 19 | 9 | 25 | 13 | 12 | 31 | | |
| 4 | 0 | 13 | 10 | 17 | 17 | 26 | | | |
| 2 | 20 | 12 | 24 | 15 | 5 | 15 | | | |
| 2 | 5 | 3 | 11 | 8 | 32 | 25 | | | |
| 2 | 0 | 7 | 20 | 4 | | | | | |
| 0 | 5 | 6 | | 16 | | | | | |
| 1 | 10 | 22 | | 27 | | | | | |
| 2 | 23 | 3 | | 32 | | | | | |
| 7 | 3 | 26 | | 30 | | | | | |
| 1 | 2 | 5 | | | | | | | |
| 0 | 21 | 4 | | | | | | | |
| 5 | 0 | 17 | | | | | | | |
| 11 | 8 | 11 | | | | | | | |
| 4 | 19 | 2 | | | | | | | |
| 2 | 10 | 8 | | | | | | | |
| 12 | 6 | 14 | | | | | | | |
| 16 | 2 | 24 | | | | | | | |
| 4 | 0 | 12 | | | | | | | |
| 1 | 19 | 17 | | | | | | | |
| 5 | 2 | 17 | | | | | | | |
| 3 | 15 | 21 | | | | | | | |
| 2 | 11 | 4 | | | | | | | |
| 2 | 6 | 10 | | | | | | | |
| 1 | 5 | 16 | | | | | | | |
| 3 | 3 | 28 | | | | | | | |
| 13 | 32 | 11 | | | | | | | |
| 2 | 17 | 1 | | | | | | | |
| 7 | 2 | 14 | | | | | | | |
| 1 | 0 | | | | | | | | |
| 1 | 20 | | | | | | | | |
| 12 | 3 | | | | | | | | |
| 0 | 22 | | | | | | | | |
| 4 | 20 | | | | | | | | |
| 3 | 2 | | | | | | | | |
| 4 | 9 | | | | | | | | |
| 4 | 23 | | | | | | | | |
| 6 | 17 | | | | | | | | |
| 11 | 8 | | | | | | | | |
| 5 | 10 | | | | | | | | |
| 12 | 2 | | | | | | | | |
| 3 | 0 | | | | | | | | |
| 9 | 8 | | | | | | | | |
| | 3 | | | | | | | | |
| | 13 | | | | | | | | |
| | 0 | | | | | | | | |
| | 12 | | | | | | | | |
| | 0 | | | | | | | | |
| | 5 | | | | | | | | |
| | 21 | | | | | | | | |
| | 14 | | | | | | | | |
| | 28 | | | | | | | | |

